# Applying a Holistic Injury Prevention Approach to Elite Triathletes

**DOI:** 10.3390/sports12080225

**Published:** 2024-08-19

**Authors:** Héctor Arévalo-Chico, Sergio Sellés-Pérez, Roberto Cejuela

**Affiliations:** Physical Education and Sports, Faculty of Education, University of Alicante, 03690 San Vicente del Raspeig, Spain; hector.arevalochico@ua.es (H.A.-C.); roberto.cejuela@ua.es (R.C.)

**Keywords:** multifactorial injury prevention, endurance training, strength training, triathlon

## Abstract

(1) Background: Studies on injury prevention programs are lacking for triathletes. The aim of the present study was to describe the results of a holistic (injury) training prevention program (HITP), based on training load control and strength training, in elite triathletes. (2) Methods: The study was conducted over 2021–2023 and involved 18 males and 10 females from the same training group. The HITP itself included various methods of fatigue monitoring, strength training focused on the prevention of overuse injuries (OIs), cycling skills training, and recovery strategies. The total number and type of injuries that were sustained, subsequent training/competition absence time, and injury incidence were determined. (3) Results: Twenty-four injuries were recorded over all three seasons, i.e., 0.65 injuries per 1000 h of training and competition exposure. Fourteen injuries were traumatic injuries (TIs) and ten were OIs. Of the OIs, four were of minimal severity, two were mild, three were moderate, and one was severe (accounting for 1–3, 4–7, 8–28, and >28 days of training absenteeism, respectively). A total of 46.4% of the participants did not present any type of injury and 71,4% did not incur any OIs. Average absenteeism was 17.3 days per injury. (4) Conclusions: The HITP design and implementation resulted in low OI and severe injury incidence. Due to their unpredictable nature, the number of TIs was not reduced. The TIs were suffered more frequently by men. Women are more likely to suffer from OIs, so it is particularly important to prevent OIs in women.

## 1. Introduction

Triathlon is an Olympic sport in which swimming, cycling, and running are timed consecutively. The most common distance in the short-distance triathlon is the so-called Olympic distance (i.e., 1.5 km swimming, 40 km cycling, and 10 km running) [1].

Triathletes are subjected to high training loads when preparing for competition because of the multidisciplinary nature of the sport. A professional triathlete racing in the elite category may average weekly training volumes of 15–27 h over the course of a full season [2,3]. Triathlon may present lower sport-related injury (SI) incidence than several other risk or contact sports [4], but high training loads can result in frequent overuse injuries (OIs) or traumatic injuries (TIs) [5,6,7]. Previous studies have indicated that injury prevalence during the competition phase ranges from 2% to 15% [8]. Vleck et al. [9] showed that the most common types of SIs among high-level triathletes are OIs (with 72% reporting at least one) followed by TIs (with 43% of athletes reporting at least one). Moreover, the most frequent anatomical location of injuries is in the lower limbs [7,10]. This is due to the high impact of the run, the exercise mode in which most injuries occur [7].

SIs are a major concern for coaches [11]; not only do they affect the athlete’s health, but, because they impede training, they can also lead to poorer performance. 

This is why SI prevention should be regarded as a key component of an effective training plan. However, to the best of our knowledge, no studies have hitherto presented any specific injury prevention programs for triathletes [5,6,7,8,12,13]. The nature of SIs in triathlon is multifactorial and complex, prompting a holistic prevention approach [14,15]. In such prevention programs, knowledge of risk factors must be translated into real training contexts [15]. The fundamental aspects of a holistic injury training prevention program (HITP) would include systematic strength training; indeed, a recent systematic review and meta-analysis demonstrated that a 10% strength training volume increase reduces SI risk by over four percentage points [16]. Nonetheless, triathletes often overlook strength training, owing to misconceptions about the use of concurrent training as well as because of practical considerations [17]. The adequate monitoring of training load is a second important aspect of a successful HITP. Proper management of both internal loads (e.g., rating of perceived exertion (RPE), heart rate) and external loads (e.g., training duration and intensity) helps balance performance enhancement and injury risk. Research shows that sudden changes in training load and excessive or insufficient training can significantly increase injury rates [15,18]. Therefore, systematic monitoring and adjustment of training loads are essential for optimizing performance and reducing the risk of injury [19]. Other techniques such as deep tissue massage and myofascial release not only enhance muscle recovery but also improve flexibility and circulation, thereby reducing injury risk. Consistent application of massage therapy has been shown to prevent common sports injuries by maintaining muscle integrity and optimizing an athlete’s overall functional capacity [20]. Suarez et al. [21] provide a good illustration of an HITP involving these topics. They developed a program for professional football players that embraced strength training interventions, physiotherapy, load control, and constant technical staff feedback. The aforementioned HITP had important results in practice. It reduced the total number of SIs that were sustained by the athletes by more than half over the seasons during which it was implemented. 

The issue of injury has been a recurring subject in the field of sports science over the last decade. A number of descriptive and experimental studies have addressed both the causes of injury, and rehabilitation and prevention protocols [22,23,24]. However, much of such literature has focused on more popular sports, such as football or basketball [25,26]. In the domain of endurance sports, some triathlon-related studies have been published on swimming [27], cycling [28], and running [29]. They found that the implementation of injury prevention protocols (including strength, stretching, and physiotherapy) reduced OI risk. Nevertheless, to our knowledge, no studies with high-performance athletes, detailing the characteristics of the HITP and its effects on injury incidence, have been conducted in the sport of triathlon. A comprehensive description of such a triathlon-specific program that thoroughly describes all the strategies used would likely help coaches and athletes to improve their training plans. This could consequently lead to a reduction in their risk and/or severity of sports injury.

Thus, the aim of the present study was to provide an in-depth account of an HITP proposal that was implemented in high-level triathletes over a 3-year period, together with its effects.

## 2. Materials and Methods

### 2.1. Participants

A total of 28 athletes participated in the study, of whom 18 were men and 10 were women. Based on McKay’s framework for sport science research [30], of the male triathletes, 4 were categorized as tier 5 (world-class level) athletes, 6 as tier 4 (elite/international level) athletes, and 13 as tier 3 (highly trained national level) athletes. That is, 10 males were international-level and 13 were national-level triathletes. Of the female triathletes, 4 were tier 4 and 6 were tier 3. Overall, the study participants included 4 triathletes with at least one podium finish in the World Triathlon Series, 5 with at least one podium finish in the World Triathlon Cup, 9 with a top 10 finish in the World Triathlon Cup, and 9 national champions. The triathletes were of 7 different nationalities. The athletes belonged to the same triathlon team, followed the same training methodology, and were led by the same coaches (i.e., AT, HA, RC, and SS). The data presented in this work correspond to athletes who were part of the training group for at least one year over the 2021, 2022, and 2023 seasons.

As regards the male triathletes, their mean and standard deviation (SD) for age, height, and weight were 22.1 (4.2) years, 179.3 (8.7) cm, and 68.2 (5.3) kg, respectively. For the women, average age, height, and weight were 20.7 (4.6) years, 169.3 (7.5) cm, and 58.2 (4.1) kg, respectively. All the study participants had over 5 years of experience in triathlon training and competition. They underwent a medical check-up at the start of each season to confirm their physical readiness for intense exercise. The study participants also gave written informed consent in advance for their data to be used in the study. The study procedures were approved by the Ethics Committee (Expedient UA 2022-09-29_1) of the University of Alicante and followed the data collection guidelines of the Declaration of Helsinki.

### 2.2. Study Design

The present study was a longitudinal prospective descriptive study. The data were collected over the course of three entire calendar years, in the years 2021, 2022, and 2023. 

The triathletes performed an HITP that was itself divided into 5 different sections (i.e., strength training, training load control, session development control, bike skills training, and physiotherapy treatment), all of which were worked on continuously throughout the season. All the interventions were coordinated with each other. Effective communication between the staff and medical team was strongly focused on throughout, with the athletes’ health being regarded as the priority.

### 2.3. Injuries and Exposure

Only SIs that were attributed to triathlon training or competition were recorded. SI diagnosis and treatment were performed by the medical team. In more severe SI cases, the clinical diagnosis was verified via magnetic resonance imaging. A record book was used to take note of SI type, its anatomical location, and the (subsequent) duration of sports-related inactivity. 

In line with previous studies [5,7,31], SIs causing absence from participation in at least one training session of any discipline were considered as recordable. Depending on the underlying injury mechanism, the SIs were categorized either as TIs (e.g., due to a bike crash) or as OIs (e.g., stress fracture). The SIs were categorized by anatomical location, as either lower body or upper body injuries. Injury recovery was considered complete when the participant was able to return to training in all three triathlon disciplines. SI severity was determined by the duration of the interval between the date on which the injury occurred and the triathlete’s return to full training. Injury severity was categorized as follows: minimal (1–3 days of missed active participation), mild (4–7 days missed), moderate (8–28 days missed), or severe (over 28 missed days) [21]. 

Injury rate (IR) was determined as the number of total injuries that were recorded per 1000 h of training and competition. To calculate training and competition volume, all the study participants recorded their triathlon training and competition sessions using their training recording devices. The specific training analysis instrument that was used for monitoring endurance sports was the COROS Training Hub software (COROS Wearables Inc., Irvine, CA, USA).

### 2.4. Intervention

The HITP was implemented in the same way for all the study participants throughout each of the three seasons for which the study ran. Priority was given to some of the topics of the HITP depending on the season period, as illustrated in Table 1. 

#### 2.4.1. Strength Training

**Figure 1:**
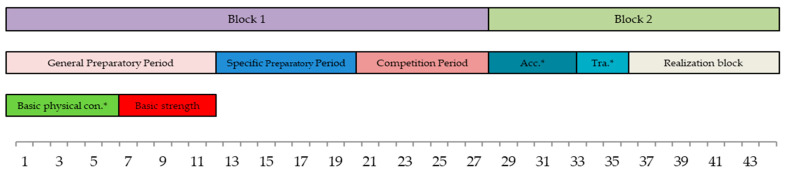
Timeline of the strength plan. * Acc = accumulation; Tra = transformation; Con = conditioning.

The strength training planning was structured based on the athlete’s schedule of competitions and training sessions (Figure 1). Each season was planned according to each athlete’s individual competition calendar. Due to the characteristics of the athlete’s competitive calendar, a first training block of traditional-style periodization, followed by a second phase of block periodization (ATR style), was implemented [32]. The first, traditional periodization block of the season was the longer of the two blocks. The traditional periodization model can be useful for athletes who do not have an excessive number of competitions during this period. The athletes can benefit from a multi-task training program by developing several capacities and abilities at the same time, while the accumulated fatigue and any negative training transfer effects that can occur between such capacities are taken into account [32]. The second block was significantly shorter. In view of the residual training effect of the first block, it was decided to employ an ATR model for this second block of the season. Given the increased number of scheduled competitions within the aforesaid period, it was necessary for the athletes to work on different skills over a shorter period of time. The ATR model was deemed the most suitable model under such conditions [32]. The introduction of several methods of strength training was proposed because studies have shown that endurance athletes improve their strength levels and economy more with strength training programs that contain several methods (e.g., plyometric and maximum strength training) as opposed to a single method [33].

A submaximal load–velocity profiling test [34] was realized to estimate the one repetition maximum (1RM) of each triathlete, and then to set his/her individual loads on the basis of this, for each of the basic strength exercises that were utilized by the program.

**Figure 2:**
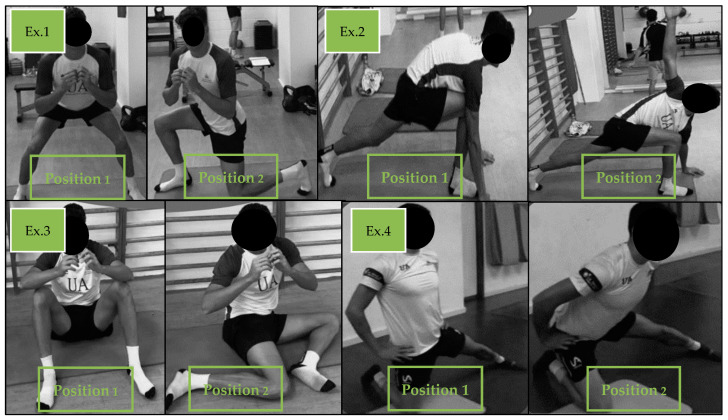
Examples of different exercises for hip mobility training.

- Block I (traditional-style periodization):

This period lasted 28 ± 3 weeks and included the entire general preparatory period (of 12 ± 2 weeks), the specific preparatory period (of 8 ± 1 weeks), and the competitive period (8 ± 2 weeks). During the general preparatory period, a first, basic physical conditioning phase, which involved 4 weekly sessions, unfolded for 6 weeks. The sessions focused on developing endurance strength using low loads (40–60% RM), with a 1 min rest period between sets. They involved two different types of (1) unilateral exercises; (2) complementary mobility hip and scapula exercises; and (3) complementary strengthening exercises of the core, Achilles tendon, tibial muscles, peroneal muscles, as well as of intrinsic foot and gluteus muscles. Figure 2 shows an example of hip mobility exercises. In this phase, isometric strength “Iso-Hold” hip-type exercises were also performed in 2–3 sets of 2–3 repetitions, lasting from 10 to 30 s on each side [35]. This training was conducted at the beginning of the season to generate a general muscular physical conditioning in the athlete, to avoid muscular decompensation, and to work on the stabilizing musculature. This should allow for the accumulation of more training load, as well as for strength training with higher loads [32,36].

**Figure 3:**
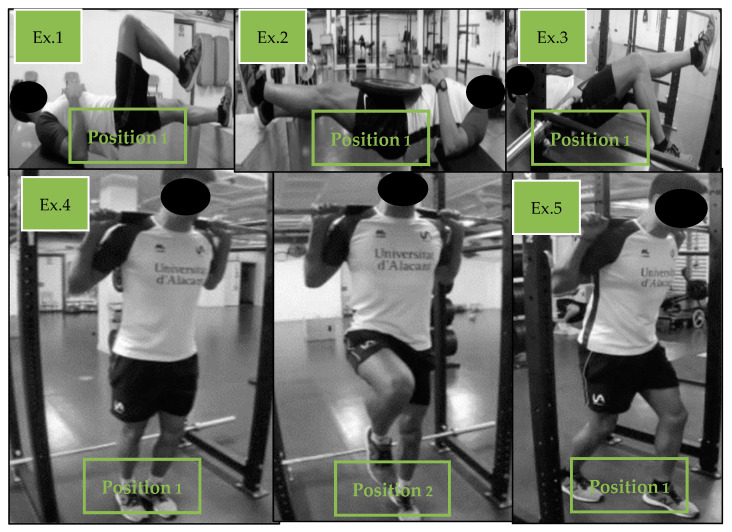
Examples of different exercises for iso-hold and iso-push hip training.

The second phase of the general preparatory period was the basic strength development phase, which lasted 6 ± 1 weeks. In this phase, 3 sessions were performed per week. The workload that was performed was between 65 and 75% of 1RM, in 3–4 sets of 5–8 repetitions and with a 3′ rest between sets, i.e., of medium–low-level effort. This design was implemented so as to produce maximum strength improvement while avoiding the development of excessive fatigue that could then affect the athletes’ main endurance training sessions. In this way, we avoid the excessive accumulation of metabolites and muscle damage, as well as the generation of hypertrophy, which is unnecessary for endurance sports [33,37,38]. The exercises that were performed were a total of 5 multi-joint exercises, divided into upper body (pull-ups and bench press) and lower body (deadlift, hip thrust, squat, and Bulgarian squat) exercises. In each session, 2 lower body exercises were alternated with one upper body exercise. There were no exercise repetitions in consecutive workouts. A reduced range of exercises was chosen so as to ensure technique mastery and to optimize the use of training time [39]. Once they had mastered the exercise technique, the triathletes were encouraged to move the load as quickly as possible, so as to generate adaptations at the neural level. This high-velocity movement training has been shown to improve high-velocity athletic performance, particularly among well-trained athletes [40]. During this phase, the athletes continued to perform the same “hip Iso-hold” work and introduced “Iso-push” exercises in 2–3 sets of 2–3 repetitions of 3 s on each side with 1 min rests between sets. Figure 3 shows examples of the “Iso-hold” and the “Iso-push” exercises.

**Figure 4:**
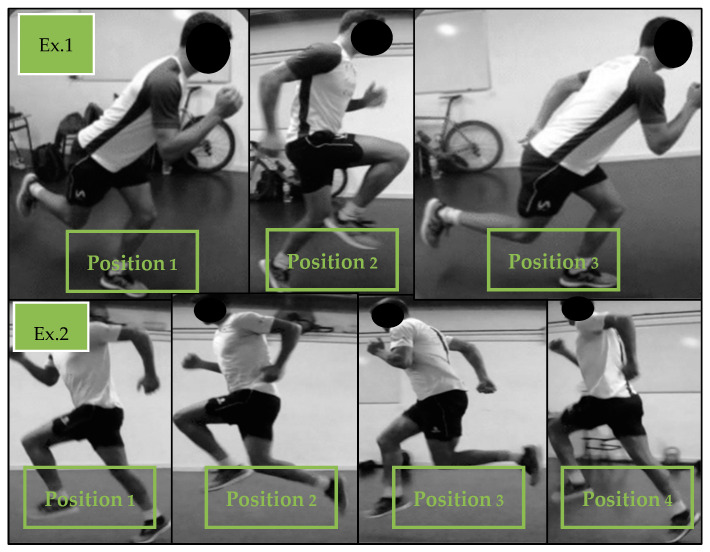
Examples of different exercises for lower body contrast training.

**Figure 5:**
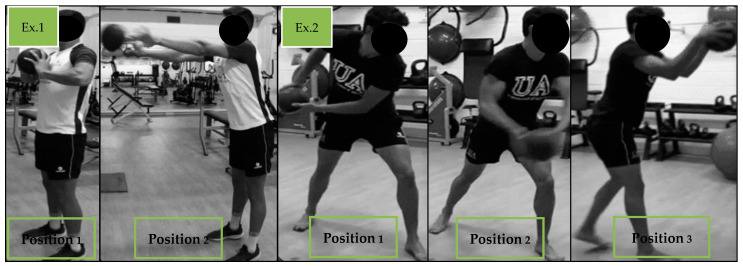
Examples of different upper body throw exercises for contrast training.

During the specific preparatory period, training frequency was 2 sessions per week. The exercises that were performed were identical to those that had been implemented within the previous training phase. The load was 60–70% of the athlete’s 1RM, in 2–3 sets of 4–6 repetitions each. In this phase, “contrast training” was included; following the upper body strength series, medicine ball throwing exercises were performed (4–6 repetitions), and after the lower body exercises, hurdle jumps or box jumps were carried out (4–6 repetitions). Figure 4 and Figure 5, respectively, show examples of the upper body contrast exercises and lower body contrast exercises that were utilized. Over this period, the “Iso hold” work was halted, and the “Iso-push” work was maintained, with the load for the latter being increased to 3–4 sets of 3–4 repetitions of 3” on each side. The contrast method has been shown to generate positive effects on both absolute strength gain and running economy [35].

In the competitive period, strength training frequency was reduced to 1 session per week. The same basic strength development sessions were performed along with contrasts. The number of sets was reduced to 1–2, while the number of repetitions and loads was maintained as before. “Iso-pushes” exercises were only performed as contrast training.

- Block II (ATR periodization)

This period lasted 16 ± 2 weeks and comprised a first accumulation period (of 5 ± 1 weeks’ duration), a (3-week) transformation period, and a realization period (lasting 8 weeks). During the accumulation period, the training protocol that corresponded to the basic strength development phase of the general preparatory period was repeated. In the transformation period, the protocol of the specific preparatory period was repeated. In the performance period, the competitive period protocol of block I was repeated.

Throughout the season, complementary strength work was maintained on at least 3 days per week. Hip and ankle mobility exercises were carried out to warm up before running training sessions. Scapular mobility exercises were also performed before any swimming training sessions. The same periodization was implemented for the female triathletes as for the males, apart from the fact that that they performed one more upper body exercise in all the basic strength development sessions.

The objective, in so doing, was to increase work volume and to thus minimize the strength differences that existed between the upper and the lower limbs (this being accentuated in women [41]. The females were also given a larger volume of supplemental training than were the males, in order to minimize the occurrence of overuse injuries [24].

#### 2.4.2. Training Load Control

To calculate and control the training load, the Objective Load Equivalent (ECO) methodology was employed. To summarize, based on laboratory performance tests with cardiorespiratory and lactate analysis, 8 intensity zones were defined for swim, cycling, and run. These zones were correlated with an individual RPE scale ranging from 1 to 10 [42]. Training load was determined by multiplying the duration (in minutes) of training time that was spent in each training zone (1–8) by a score value that ranged from 1 to 50 (and was based on that training zone), and then by applying specific weighting factors for running, swimming, and cycling. Subjective Load Equivalents (ECS) were also assessed, allowing athletes to subjectively quantify session difficulty on a scale of 0 to 5. The ECOs were calculated using the All in Your Mind Training 143 system^®^. Subjective data such as hours of sleep and ECS were recorded by the athletes in online training logs.

Special attention was paid to maintaining a balance in training loads, by alternating development sessions and recovery sessions. Generally, in an average training week, Tuesdays, Wednesdays, Thursdays, Saturdays, and Sundays were dedicated to developing different abilities depending on the time of the season, while Mondays and Fridays were active recovery days. The training load was reduced on recovery days, so as to avoid the development of states of excessive fatigue that could themselves lead to injury [42]. 

As a rule, the weekly training load was increased by 10–15% compared to the previous week in the development microcycles, and decreased by 20–25% in the recovery microcycles of the program. An undulating periodization model was used during the competition period. The training load was reduced compared to previous periods, maintaining intensity but lowering volume [42]. 

The athletes and the coaches were in continuous communication with each other throughout the season. If an athlete recorded excessive ECS values or transmitted subjective feelings of excessive fatigue, the training load was reduced to avoid states of non-functional overreaching that could lead to injuries.

#### 2.4.3. Session Development Control

To monitor their adherence to session intensity and goals, the athletes were asked to remember and respect the intensity zones and their associated speed/power, heart ranges, and RPE. Low-intensity workouts (zones 1 and 2) were mostly guided by heart rate and RPE, while speed and power were used to regulate sessions of moderate to high exercise intensity (zones 3–8).

At least one coach (i.e., AT, HA, RC, or SS) was present to monitor the session in all training sessions. Additionally, the participants recorded their training sessions using training devices. The COROS Training Hub software (COROS Wearables Inc., Irvine, CA, USA) facilitated the control and monitoring of the athletes’ training by their support team.

#### 2.4.4. Cycling Skills Training

During the general preparatory period, the emphasis was on the refinement of fundamental skills that are essential for overall cycling proficiency and safety. A weekly, technique-only training session was conducted. This technical session was designed to enhance various cycling skills, including riding in a group, cornering, the ability to do U-turns, mounting and dismounting the bike, and other bike handling skills. 

In the specific preparatory period, the athletes’ bike skills were further improved via the use of brick (bike and run) sessions, once a week. The brick sessions took place on a closed circuit and involved 3 curves per kilometer. These sessions were carried out in groups, and close to or at exercise intensities resembling that of competitions, so as to replicate actual competition conditions as closely as possible. By simulating race-like conditions and incorporating targeted skill development, athletes were primed to optimize their performance and confidence in competitive events. It is also possible to conduct some group cycling training at high speeds, practicing skills such as draft relays. These exercises replicate the movements that occur within competitive cycling groups. Within the peloton, positions are alternated to achieve higher speeds more efficiently.

#### 2.4.5. Physiotherapy

The athletes worked with the physiotherapist throughout the season and this collaboration played a key role in the HITP program of each triathlete. As a rule, one physiotherapy session took place per week. Some athletes had two sessions per week during peak season and these sessions were carried out on their recovery days. Myofascial induction techniques were performed to readjust the connective tissue mechanical properties and to facilitate drainage and recovery. In the case of injury having occurred, the medical team carried out the diagnosis. They designed the specific injury treatment and readaptation plan that was to be implemented for the injury in question jointly with the physiotherapist.

### 2.5. Data Analysis

A descriptive analysis was conducted using the means and standard deviation of the variables that were recorded within the study. Injury rate was calculated as the number of injuries per 1000 h of triathlon training and competition. The analyses were performed using a Microsoft Office Excel 2016 spreadsheet.

The nonparametric Mann–Whitney U test was performed to detect the statistical differences between the variables for men and women. Spearmans’ bivariate correlation coefficient was used to determine the existence of any inter-relationships between the occurrence of injuries and the time of exposure and the years of experience performing HITP. The statistical software Statistical Package for Social Sciences (SPSS) version 22.0 (SPSS Inc., Chicago, IL, USA) was used to analyze the data. For all analyses, significance was accepted at *p* < 0.05.

## 3. Results

**Figure 6:**
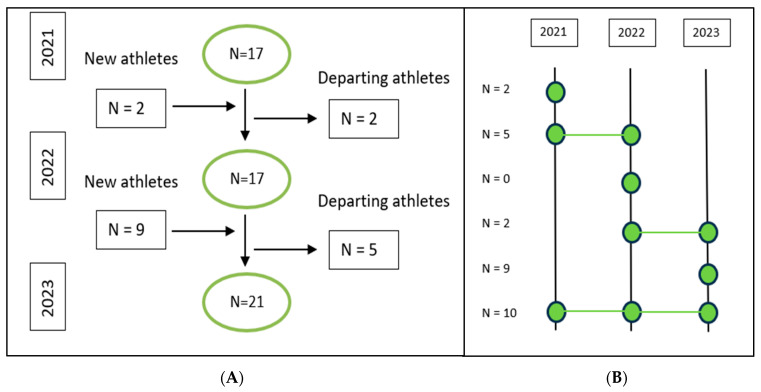
(**A**) The number of new and departing athletes each season. (**B**) The number of athletes in the training group across each season.

A total of 28 triathletes (10 females, 18 males) were prospectively followed across the three consecutive years of the study (Figure 6). The number of participating male athletes was 11, 12, and 14 for 2021, 2022, and 2023, respectively. A total of six, five, and seven female athletes took part in 2021, 2022, and 2023, respectively. Seven male and three female athletes were present throughout all 3 consecutive years of the program.

Mean and standard deviation (SD) participant exposure per calendar year was 660 (122) hours. The men’s exposure was slightly higher (671 (33) hours) than that of the women (649 (89) hours), but not significantly so. Table 2 presents the corresponding injury data of the athletes. During the recording period, 24 SIs occurred overall, across all 28 study participants. Ten injuries (41.6% of the total SIs) were OIs and fourteen (58.3% of the total SIs) were TIs. The number of TIs that were incurred was significantly higher among the men than among the women (i.e., twelve TIs for the men vs. two for the women). Of the 28 athletes that participated in the study (i.e., regardless of the number of years that they participated in the program), the overall percentages of athletes affected by injury were 53.6% for any kind of injury, 28.6% for OI, and 35.7% for TI. In the males, the percentage of athletes affected by injury was 55.6% for any kind of injury, 27.7% for OI, and 44.4% for TI. The corresponding values for the male participants recorded 30% for any kind of injury, 30% for OI, and 20% for TI. A statistically significant difference between the sexes was found in the percentage of athletes who suffered a TI. Among the ten athletes who took part in all three years of the study, the highest percentage of athletes being affected by injuries was noted in 2021 (i.e., 50%). In 2022, this value decreased to 30%. The lowest percentage of athletes being affected by injuries was noted in 2023, at 20%. 

When, even if the athletes in question did not take part in all three years of the study, the number of years of experience of the HITP program was considered, the highest percentage of athletes affected by injuries was recorded during the first year of performing the HITP (e.g., 35.7%, n = 28). In the second year, this value decreased to 31.6% (n = 17). The lowest percentage of athletes affected by injuries was recorded in the third year of performing the program, with 20% affected (n = 10). 

In terms of injury severity, significant differences were found between the sexes for the number of moderate-severity injuries that were sustained, with the men recording six and the women one. For the injuries that did occur, average and standard deviation (SD) absenteeism was 17.3 (54.2) days. Statistically significant differences were found between the men’s absenteeism and women’s absenteeism as a result of injury. This was 22.6 (55.6) days and 5.83 (8.7) days for men and women, respectively (*p* < 0.05). The registered IR during the recording period, which did not differ with sex, was 0.65 injuries per 1000 h overall. This equated to 0.68 injuries per 1000 h of training and competition exposure for the men and 0.61 injuries per 1000 h of training and competition exposure for the women. IR due to trauma was higher in men than it was in women (at 0.46 vs. 0.2 injuries per 1000 h of training and competition exposure, respectively). The IR for overuse injury was higher in women (at 0.41 injuries per 1000 h of exposure) than it was in men (at 0.23 injuries per 1000 h of training and competition exposure) but the difference was not statistically significant.

A small but statistically significant negative correlation was noted between the number of years that participants had been involved in the HITP program and the number of injuries they experienced during the year (ρ = −0.395; *p* < 0.05). No such statistically significant correlations were found for TI (ρ = −0.034; *p* > 0.05). No statistically significant correlations were found between exposure time in training and competition and the number of OIs (ρ = −0.22; *p* > 0.05) or TI (ρ = 0.14; *p* > 0.05) that were sustained by the athletes.

## 4. Discussion

The study objectives were to present an HITP proposal together with the results of its implementation over a 3-year period, in high-level triathletes. The main findings were that the implementation of an HITP resulted in low injury incidence (and particularly overuse injury incidence). Among the ten athletes who participated in all three years of the study, the percentage of athletes who were affected by injury decreased over the course of the three consecutive years. We also noted a small, but statistically significant, negative correlation between the individual athletes’ years of experience of participation in the HITP and the number of overuse injuries that were suffered during the season by the study participants. Nevertheless, the most frequent kinds of injury that occurred were traumatic injuries. Men showed significant differences in the number of traumatic injuries that were incurred over the course of the study compared to women. Women presented a larger number of overuse injuries compared to males, but the difference was not statistically significant. Most of the injuries that occurred were of mild or minimal severity. 

Over 2021–2023, i.e., the three years that the present study lasted, 53.6% of the participants (regardless of the years they participated in the program) suffered at least one sporting injury. These values are lower compared to the limited information that is available on this topic from previous prospective studies of elite triathletes. Vleck et al. [15], in a seven-month longitudinal prospective study of 71 members of the 1996 Great Britain National and Scottish National Triathlon Squads, noted a total of 80.4% of athletes that were affected by injury (regardless of severity). Crunkhorn et al. [43], in their four-year, prospective study of 50 Australian national elite squad triathletes, reported 92% of the athletes to have been injured over 2018–2021. It is important to note that this study, unlike the present one, also noted non-time loss injuries. In the Crunkhorn et al. [43] study, 67.3% of the injuries resulted in a period of time loss. We note, however, that differences in methodologies complicate the comparison between studies [7].

As regards the ten triathletes who participated in all three years of the HITP program, the highest percentage of those who were affected by injuries occurred in 2021 (e.g., 50%). This percentage dropped to 30% in 2022, and then to its lowest value in 2023, with 20% of athletes affected. Based on the years of experience in the program, it can be observed that during the first year performing the HITP, 35.7% of the overall cohort of 28 athletes suffered at least one injury. This percentage decreased in the next year for the 17 athletes who continued in the program for a second year (i.e., 31.6%). Among the 10 athletes that performed the program for three consecutive years, in the third and last year, this value was 20%. It can be observed that by implementing an HITP, the percentage of athletes who suffered an injury seems to decrease each year. HITP experience may enhance the injury prevention capacity of the program. This may be due to the adaptations that are generated year after year. It may also be due to the increased ability of athletes to perform the program successfully. 

A higher number of lower body SIs were also recorded in the present study, which is consistent with most literature results [10,44] and is probably due to the impact generated by the run.

The injury incidence rate in this study was 0.65 injuries per 1000 h of training and competition exposure. This value is also lower than that of previous studies on elite triathletes, which reported values of between 17.5 and 58.1 per 1000 h of training exposure in seven consecutive months [15]. However, this comparison may not be completely correct because of the different data registration periods of the two studies. Unlike the present study (which collected data throughout the year), Vleck et al.’s [15] investigation was carried out between the months of February and August, when traditionally there is more training load and competitions. This may have increased the injury risk. In running, injury incidence has been reported to vary between 2.5 injuries per 1000 h of training and competition exposure (in a study of long-distance track and field athletes) and a maximum value of 33.0 injuries per 1000 h of training and competition exposure (in a study of amateur runners [29]). In the present study, no correlation was found between the training exposure time of the athletes and the amount of sporting injuries that were incurred by the triathletes. This may show the possible effectiveness of the HITP in terms of injury prevention.

In terms of injury absenteeism, an average of 17.3 days spent without being able to train in at least one discipline was reported in the present study. Vleck et al. [10], in a retrospective study, reported an average absenteeism of 29.3 days off running training for national squad athletes who were racing non-drafting competition. However, Vleck et al. [15], in their prospective seven-month study on elite triathletes, found an average length of time that injury lasted between 12.8 days and 5.6 days, depending on the area affected by the injury. On the other hand, Crunkhorn et al. [43] noted an injury burden calculated across the 4-year surveillance period of 68.39 days of time loss per 365 days. Again, the differences in the study design complicate the comparison between studies.

Regarding the origin of the SI, we can observe that in the present study, the percentage of athletes who were affected by injury (at 28.6% and 35.7% for OI and TI, respectively), the IR (at 0.27 and 0.38 injuries per 1000 h of training and competition exposure for OI and TI, respectively), and the amount of injuries (at 14 OI and 10 TI, respectively) were higher when the injuries were traumatic than when they were classified as being due to overuse. These injury data together with the negative relationship found between the years of experience of performing the HITP and the number of OIs that were sustained may indicate that the HITP program that was presented in this study can be an effective one, especially as regards OI. Although we cannot be sure that the effectiveness of HITP is due to strength training alone, it has been frequently shown that strength work can prevent OI occurrence [45]. Complementary strength training that focuses on higher-SI-risk joints also contributes to injury prevention [36]. The structure created for strength training can also benefit triathletes’ performance. This would be due to concurrent training, which improves the energy cost of locomotion [46]. Moreover, low to moderate strength training efforts with repetitions together with muscle failure avoidance may improve performance while also preventing the negative effects of muscle hypertrophy or fatigue [47]. In addition, isometric strength work is a good complement to the aforesaid type of training, as it increases strength without generating any major muscle fatigue [35]. The primary objective with strength training was not to maximize strength values but to reach acceptable levels to aid in injury prevention. The combination of various strength training methods may also have influenced the acquisition of optimal strength levels that minimize the occurrence of injuries [33].

Another possible reason for the low injury incidence values that are reported here may be the training load and the control of session development that took place over the course of the study. Training planning errors are a common cause of SI [48], therefore, maximizing the level of control that is exerted over the design and execution of the athletes’ training plan is key to avoiding the overload that can lead to SI. The goal in this part of the HITP was not to exceed the maximum load tolerable while generating the greatest possible number of adaptations. Managing training loads based on both external and internal load data methods and mastering the intensity of training zones the athlete is familiar with can help to regulate the balance between training dose and training response. In addition, implementing identification systems of overtraining symptoms can help to prevent OI, e.g., by monitoring subjective fatigue values [49]. This latter strategy is all the more essential in periods of more intense efforts, such as in specific periods or competition periods [6]. Another key factor is regular work with the physiotherapist. Myofascial induction and sports massages have been proven to be effective methods to recover from fatigue and to prevent SI [20]. Therefore, working with a multidisciplinary team able to offer this service can greatly benefit the athletes’ health and performance.

Despite its apparently positive OI prevention results, the HITP was not found to be effective at preventing TI. We did not find a statistically significant relationship between the athlete’s experience of the HITP and the number of TIs that were incurred over the course of the study. This could point to the uncontrollable condition of TI [9]; despite working on cycling skills, athletes continue to suffer SI, mainly caused by bike segment falls. This phenomenon can also be observed among professional cyclists. Haberle et al. [28] recorded that during the 2010 to 2017 Tours de France, 53% of race dropouts were due to acute trauma and 47% to non-traumatic causes, such as OI or illness. In triathlon, the points where participants anticipate the most risk of traumatic injury are the cycling mount/dismount area and the cycling segment [6,50]. The less experienced the triathlete, the higher the risk of accidents in these sections [6,50]. Therefore, future studies should focus on developing accident prevention programs, perhaps including techniques such as imagery, which has been shown to be effective in other sports such as BMX [51].

As for gender differences, in this study, both sexes presented similar levels of injury occurrence. Although the percentage of athletes who suffered an injury between 2021 and 2023 was higher for males than it was for females, i.e., 55.6% and 30% for males and females, respectively, no significant differences were found. This finding is compatible with previous studies, which have shown the absence of a relationship between triathlon SI and gender [43,52]. However, TIs were more numerous and severe in men than in women (*p* < 0.05). This may be due to the higher speeds that are attained by men in the cycling segment, especially in competition, which can cause more severe TI if they suffer a fall [53]. However, although no statistically significant differences were found, OI IR was higher in women than in men. Women generally tend to suffer OI more frequently, possibly due to lower strength levels and hormonal causes [54]. Other studies have also pointed out that women tend to suffer more bone stress injuries than men [43]. Nevertheless, in the present study, women’s IR was much lower than that reported by Hamilton et al. [55]. A possible explanation would be the preventive effect of the female-specific strength programming that was implemented in this study by increasing the women’s strength training load compared to that of men, especially as regards that which was induced whilst doing the complementary and upper body exercises [41] (as illustrated in Figure 2).

This study has made a valuable contribution to the field, as for the first time, an injury prevention plan with potentially positive results has been proposed for triathletes. However, the work has several important limitations that should be considered when interpreting its results. The fact that we did not have a control group (either of matched high-performance athletes or of the same athletes, from before they underwent the HITP) limits the ability to directly attribute the observed injury outcomes to the HITP itself. Another important limitation of our study is the inability to identify which specific components of our training program might be most effective in reducing injury incidence. The training program was designed as a holistic approach, and the athletes who participated in it sustained low injury incidence. However, as this is a descriptive study, it does not allow for a definitive analysis of the causal relationships between program components and any observed reduction in injury occurrence or severity. Consequently, there is no concrete way to determine whether omitting certain elements of the program would result in the same or even better injury-related outcomes. The lack of uniformity in injury definitions and methodologies across existing studies also complicates direct comparisons with the published literature. This underscores the need for further research to isolate and examine the effects of specific training interventions within the holistic framework. Moreover, implementing an HITP of this nature presents significant logistical challenges. The complexity of the plan requires considerable investment in time and financial resources, which may not be feasible for all levels of triathletes. 

Nonetheless, this study provides a concrete example of how an HITP minimizing injury incidence may be designed and implemented. We must remember, nevertheless, that injury etiology is multifactorial and such a program may not work in contexts other than the one that was here described. The question remains as to whether different approaches would improve the results.

Despite these limitations, our findings suggest that by applying appropriate adjustments and adaptations, the plan can potentially improve triathlete health and performance, offering a solid foundation for future research and practical applications in triathlon.

## 5. Conclusions

The present study presented a detailed triathlon HITP and the results of its implementation. Total SIs and severity reported were low, possibly due to the joint effect of strength training, load and session development control, and physiotherapy. A significant (but low) relationship was found between years of experience of the HITP and the number of OIs, but not of TIs, that were incurred. Though OI incidence was lower, TI did not appear to have been reduced despite the skills development work within the HITP that aimed to prevent or lessen it. In triathlon, external factors such as the course or competitor interaction make it more difficult to prevent TI. Men suffered more numerous and severe TIs than women, whose major cause of injury was overuse. There is also a need for new studies with control groups that can show what it is more effective in preventing SI in triathletes.

To our knowledge, this study is the first to present a detailed HITP proposal for a group of elite triathletes. As such, this study is particularly useful for coaches and athletes. This paper provides practical information on how to potentially minimize the occurrence of SI. Such information is important given that SI can potentially affect the health and performance of any age or level of triathlete.

## Figures and Tables

**Table 1:** Order of priority per section of the holistic training prevention program during the season.

		Strength Training	Training Load Control	Session Control	Physiotherapy Treatment	Bike Skills
Traditional periodization	General preparatory Period	****	***	****	**	***
	Specific Preparatory Period	***	****	****	****	****
	Competition Period	**	**	***	***	***
Block periodization	Accumulation Block	***	***	***	*	**
	Transformation Block	**	****	***	***	***
	Realization Block	*	**	**	**	**

* = Degree of importance of the topic from 1 to 4 stars, with a 4-star rating being the most important.

**Table 2:** Injury data and comparison between male and female triathletes.

	Overall (n = 28)	Male (n = 18)	Female (n = 10)
Training and Competition Exposure per season			
Total (mean hours (SD))	660 (122)	671 (33)	649 (89)
Injuries during the three seasons			
Total (n)	24	18	6
Overuse (n)	10	6	4
Traumatic (n)	14	12 *	2 *
Absenteeism (mean days (SD))	17.3 (54.2)	22.6 (55.6) *	5.83 (8.7) *
Overall % of athletes affected by injury			
Among all participants (n = 28)	53.6	55.6	30
In first year of following the HITP (n = 28)	35.7	38.9	30
In second year of following the HITP (n = 17)	31.6	30.8	33.3
In third year of following the HITP (n = 10)	20	28.6	0
by OI, among all participants (n = 28)	28.6	27.7	30
by TI, among all participants (n = 28)	35.7	44.4 *	20 *
Overall Injury Severity, 2021–2023			
Minimal (n)	10	6	4
Mild (n)	6	4	1
Moderate (n)	6	6 *	1 *
Severe (n)	2	2	0
Overuse Injury Severity 2021–2023			
Minimal (n)	4	1	3
Mild (n)	2	1	0
Moderate (n)	3	3	1
Severe (n)	1	1	0
Traumatic Injury Severity, 2021–2023			
Minimal (n)	6	5	1
Mild (n)	4	3	1
Moderate (n)	3	3	0
Severe (n)	1	1	0
Injury rate/1000 h of training and competition			
Overall	0.65	0.68	0.61
Overuse	0.27	0.23	0.41
Traumatic	0.38	0.46 *	0.2 *
Anatomical location of injury			
Upper body (n)	2	2	0
Lower body (n)	22	16	6

* = sex group statistically significant difference. (*p* < 0.05); OI = overuse injury, TI = traumatic injury.

## Data Availability

The data that support the findings of this study are available from the corresponding author upon reasonable request.
